# “Health inequalities in Armenia - analysis of survey results”

**DOI:** 10.1186/1475-9276-11-32

**Published:** 2012-06-13

**Authors:** Tamara Tonoyan, Lusine Muradyan

**Affiliations:** 1 Yerevan State Medical University (YSMU), 2 Koryun str., Yerevan, 0025, Republic of Armenia; 2 Department of Health Policy, Economics and International Relations at the National Institute of Health, Ministry of Health, Republic of Armenia (NIH MH RA), 49/4 Komitas Av., Yerevan, 0051, Republic of Armenia

**Keywords:** Health, Health care utilization, Health accessibility, Health inequality and equity, Health policy

## Abstract

**Introduction:**

Prevailing sociopolitical and economic obstacles have been implicated in the inadequate utilization and delivery of the Armenian health care system.

**Methods:**

A random survey of 1,000 local residents, from all administrative regions of Armenia, concerned with health care services cost and satisfaction was conducted. Participation in the survey was voluntary and the information was collected using anonymous telephone interviews.

**Results:**

The utilization of health care services was low, particularly in rural areas. This under-utilization of services correlated with low income of the population surveyed. The state funded health care services are inadequate to ensure availability of free-of-charge services even to economically disadvantaged groups. Continued reliance on direct out-of pocket and illicit payments, for medical services, are serious issues which plague healthcare, pharmaceutical and medical technology sectors of Armenia.

**Conclusions:**

Restructuring of the health care system to implement a cost-effective approach to the prevention and treatment of diseases, especially disproportionately affect the poor, should be undertaken. Public payments, increasing the amount of subsidies for poor and lower income groups through a compulsory health insurance system should be evaluated and included as appropriate in this health system redesign. Current medical services reimbursement practices undermine the principle of equity in financing and access. Measures designed to improve healthcare access and affordability for poor and disadvantaged households should be enacted.

## Introduction

The socio-economic decline following the collapse of the Soviet Union had a very drastic impact on the Armenian healthcare system. The vain attempts of healthcare reform have been hindered by a number of economic challenges. These severe social-economic conditions and the failed efforts to implement a state medical insurance program have caused a decrease in subsidized health services and utilization [1]. Low purchasing power, absence of state medical insurance, introduction of out-of-pocket reimbursements, and an increase in unreported payments has aggravated the already deteriorating population health.

Previously it was believed that the free market could serve as a rational resource allocation mechanism for the healthcare system in Armenia. Since the mid-1990s the government has started to work on a radical program of reforms but many of these efforts, in particularly those from the South Caucasus region (Azerbaijan, Georgia), have had no effect [2]. Between 1993 and 2011 several measures were undertaken towards structural and financial reforms of the health system, which led only to partial improvement, but produced some unexpected results.

Public financing of the Armenian health system has increased in relative terms in recent years and from 2000 to 2011 it increased from a total of approximately $17.8 million to about $173.6 million (or 62.5 billion AMD) i.e. approximately tenfold, nevertheless it is still low [3]. Moreover, as a percentage of total Government expenditure, Armenia’s public health funding fell dramatically between 2006 and 2008 - by 47%, from 9.7% of total Government expenditure to 6.6% of total public expenditure. The government expenditures on health care as a percentage of Gross Domestic Product (GDP) also remain low in Armenia (about 1.66% of GDP in 2011), among the lowest in the world, lower than in many poor nations in Africa and Asia. In 2000 it even fell to a low of 0.8% of GDP. This is in spite of the WHO that health care expenditures should not be less than 6-9%.

The physical conditions in health posts and polyclinics are often poor and staff has had little incentive to treat patients with respect. On the one hand the reform provides the patients with the right of choosing their primary physicians, as well as reduces inappropriate and overly expensive secondary medical care; however, on the other hand, the health care reform limits the patient’s rights of access to direct specialist care. If the primary physician does not agree to refer the patient to a specialist, there is no way for the patient to refer himself to the secondary setting without directly paying into it and without a letter of referral from a primary physician in all except for emergency cases. Thus, even though the public costs are greatly reduced by the reform, actual out-of-pocket costs increase since the patients who chose to bypass the primary care physician referral process, and see a specialist, will have to pay for the treatment.

The role of private health facilities is becoming more and more apparent in the whole healthcare framework of Armenia. According to a World Bank report (2010), the private health care spending was more than 1.2 times higher than public expenditures in 2009 [4]. Private facilities are recognized as much better organized, ensuring a higher quality of services, and familiar with the client-oriented approach and modern costing mechanisms. However, it is not clear whether private expenditures include only official payments for health care services or informal payments as well. It is also not clear if total spending on health incorporates humanitarian aid provided by international donors and the Armenian Diaspora, i.e. medicines, equipment, supplies, and professional development opportunities for practicing healthcare providers.

Along with the decrease of government’s ability to socially protect the population, the active development of a shadow market of paid medical services has been observed. The growing informal sector of the economy caused a near collapse of the old social insurance and safety nets mechanisms. Estimates of the black market health care economy in Armenia cannot be accurately estimated. According to World Bank in 2000, the share of patients making ‘informal’ reimbursements was the highest among CIS countries, reaching 91%, as compared, for example, to 74% in Russia [5]. Access to health care services has become increasingly dependent on whether a household can afford the ‘informal’ payments to providers.

The government has been trying to implement Health Insurance System for 10 years. However, in Armenia, the market literally fails to provide health insurance. At the same time a significant part of the population couldn’t afford to buy private health insurance, and current tax laws do not give incentives to the employer to provide health insurance to its employees. Besides, the social security taxes that employers currently pay for their employees’ wages and income taxes that employees pay, an additional 9% or even 3% tax on wages is not politically feasible. At this stage, given the government’s relatively low revenues, public health insurance seems to be cost prohibitive. If people are struggling for survival every day, they are less willing to pay insurance premiums in advance in order to use services at a later point in time. The low income of the population and the existence of a shadow economy make the development of public and even private health insurance very difficult.

In order to reduce the shadow turnover in health sphere in Armenia a co-payment mechanism was introduced into health care services in March 2011. It has been implemented with differentiated approach in the regions compared to Yerevan, which means that the cost for healthcare services is significantly lower in the regions. While one part of the amount was directed to increasing salaries of medical workers from about $150-$200 (60.000-80.000 AMD) to about $500-$650 (200.000-250.000 AMD) depending on the hospital and number of patients, the other part of the money accumulated in the form of co-payments was intended for the improvement of the quality of healthcare services, provision of hospitals with medicine, equipment and tax payments [6]. However, the amount of the co-payments is substantial and most part of the population cannot afford to pay it.

The State Health Agency (SHA) in Armenia that was established in 1998 is responsible for purchasing services from providers through contractual mechanisms. Since then the public budget for health care is disbursed to health care providers through contracts between the SHA and the providers. Those funds are directly transferred from the Ministry of Finance (MoF). The main tasks of the SHA are: contracting health care providers for services in accordance with Basic Benefits Package (BBP), ensuring the target use of state financial resources and reimbursement of the health care providers and quality assurance. The contractual arrangements between the SHA and the providers, however, are defined relative to the health services that are provided using state property such as infrastructure and equipment. This represents an unclear arrangement since according to the existing regulation the SHA has limited ability to supervise the functioning of the contracted enterprises, and can only solve the “problems of the filed within the limits of its jurisdiction without violation of the independence of subordinate enterprises”.

Although the range of benefits nominally available to the poorest under the program of State Guarantees is greater, the SHA payments do not act as a full “catastrophic insurance” program. In general, the amounts paid to hospitals through the SHA are less than their costs, and these institutions continue to collect sizeable out-of-pocket payments from patients. In primary care, chronic disease drugs are nominally a guaranteed benefit, but primary care budgets are inadequate to cover these costs and patients continue to pay large sums out of pocket. Except for the poorest, there is no government payment for hospital care for many chronic and degenerative diseases. For this category the levels of SHA payment are below cost (50% of overall real spending), so those who must be admitted to hospital pay substantial amounts. Hence, state funding is not even sufficient to assure guaranteed free services for socially vulnerable groups [7]. In these conditions, even many representatives of these groups, whose treatment costs are covered by the state, under the pressure of unavoidable additional payments often refuse to use the free of charge medical care services guaranteed by the state. Chronic disease continues to be “catastrophic” for many households in Armenia. The expenditures by the poorest in fact exceed the amounts spent by the richest part of the population for medication. In fact, Armenians are still making significant sacrifices to obtain prescribed drugs, particularly for chronic conditions [8,9].

Despite the stability of some health indicators in Armenia in recent years many of these are still worse today than they were in 1990. The accessibility of healthcare in Armenia has clearly worsened during the researched period of time. Armenians continue to experience poor access to health care despite the sufficient number of health facilities and medical workforce. The number of patients admitted to the hospitals of the Ministry of Health in 2010 was just over a third of the number admitted in 1990, while all hospital admissions decreased by almost 50%, meaning the cost effectiveness of the hospital system has also declined [10]. Similarly, the trends in the utilization of both medical services (hospitalizations and ambulatory visits) in 1990–2010 also followed this pattern: the annual bed occupancy rate per patient has declined from 68% to 62.1% and the number of annual per capita ambulatory polyclinic visits has also sharply dropped from 7.8 in 1990 to 3.6 in 2010, i.e. was 2.2 times less than in 90s.

Between 1998 and 2010 the morbidity rate has increased. At the same time, the morbidity of population and children 0–14 years diagnosed the first time has increased. The overall birth rate from 1970 to 1990 was around 22 per 1000 population, but has since dropped to 13.8 in 2010. Between 1990 and 2000, the birth rate sharply decreased while the overall death rate comparatively increased. This can be explained by the fact that since the beginning of the transition towards a market-oriented economy, Armenia has faced a number of difficult challenges; including a major tragic earthquake of 1988, the Nagorno - Karabakh War, referred to as the armed conflict that took place from February 1988 to May 1994, a blockade enforced by Turkey and Azerbaijan since 1993 (in place now for over 19 years), an energy crisis, recession and economic collapse. This combination of events has had severe consequences. Economic decline has placed Armenian health institutions in jeopardy, hindering reforms. Gains in freedom have been accompanied by a loss of many basic economic and social services that the population had come to enjoy and expect. Thus, the population growth rate decreased along with natality rate (Table 1).

**Table 1:** Demographic changes (per 1000 population)*

**Indicators**	**1990**	**2000**	**2001**	**2002**	**2003**	**2004**	**2005**	**2006**	**2007**	**2008**	**2009**	**2010**
Birth rate	22.5	9.0	10.0	10.1	11.2	11.7	11.7	11.7	12.4	12.8	13,7	13,8
Death rate	6.2	6.3	7.5	8.0	8.1	8.0	8.2	8.5	8.3	8.5	8.5	8.6
Natality	16.3	2.69	2.5	2.1	3.1	3.7	3.5	3.2	4.1	4.3	5.2	5.2

*Source**:***National Statistical Service (NSS) of Armenia.*

The overall mortality rate has slightly increased. In 1989–1990 infant mortality has been in the range of 20 to 25 deaths per 1,000 live births [11], lower than the rates in many of the new independent states, but higher than the values reported for developed countries. For instance, infant mortality in the United States is 9.8 per 1,000 live births [12]. According to the State of the World’s Children 2009, in Armenia, roughly 22 infants per 1,000 live births die before their first birthday. Approximately 80% of these deaths are during the first 28 days of life – the neonatal period [13]. According to the official national statistical report from 1990 to 2010, infant mortality rate (IMR - deaths per 1,000 live births) in Armenia has declined from 18.5 to 11.4 [14]. Mortality rate per 1.000 live births among children under 5 years of age comprised 13.4 per thousand in 2010, as compared to 23.8 per thousand in 1990. While other sources prove it, that the figures are different for the same period of time: IMR has declined from 46.1 to 17.5, the value for child mortality rate under-5 (per 1,000) declined from 54.5 to 19.6, that was a minimum value over the past 35 years (a maximum value of 98.3 in 1975) [15,16] and the value for neonatal mortality rate (per 1,000) declined from 26 to 11. The ratio of maternal mortality (MMR) per 100.000 live births, in 1990 was 40.1. Meanwhile, the maternal mortality rate has increased by 12.4% from 1990 to 2000 due to the large number of unassisted home deliveries and abortions caused by the infrequently use of contraceptives. Since 2000 to 2010 it has declined from 52.5 to 8.9 [17]. Maternal mortality in Russia and other former USSR countries has been also high, probably reflecting the use of abortion as the most common method of family planning - an average of five abortions per woman [18].

With considerably low public funding Armenia managed to ensure relatively good life expectancy compared to many countries in the Europe and Central Asia (ECA). Life expectancy at birth has remained high - estimated at 71 years for men and 78 years for women during 2010. Both indicators were higher than those of 1990. It is difficult to adequately explain the data, because from 1990 to 2011 the value of this indicator increased. During the Soviet era, Armenia had one of the best developed health care systems in the Soviet Union (SU) [19]. Life expectancy, which in the early 1980s was the highest in the Soviet republics (73 years), fell in the early years after independence (71 years in 1991). Since the mid-1990s, this factor has been climbing steadily and reached 73.2 in 2011 [20]. Certainly, the economic collapse impacted health system outputs, and in particular, life expectancy, but the outcomes from this may be distributed over longer periods of time, and may be felt to some extent in the future.

### Armenian household health survey results of 2006–2010

The household survey implemented by the USAID Primary Health Care Reform (PHCR) in 2006, revealed that 39.5% of sick people did not seek medical treatment because of financial constraints. In fact, 42.8% of sick people who were eligible for state guaranteed free-of-charge health care services didn’t, because state funding was far less than the real cost of treatments. Moreover, many patients who were eligible for free outpatient drugs were forced to purchase medications in the market versus state funded pharmacies due to lack of supply. Lastly, self-care was found to be 36.2% and of these people, 95.3% did not visit a doctor due to financial constraints. The study provided some answers behind the population’s morbidity, defined as not seeking medical care and lack of access, reveals the following important feature of population’s health care related behavior. The indicator for not meeting with a doctor while needing medical care is significantly lower for children. The remote distance and time was a hindering factor to visit a doctor for only 1.2% of respondents and 3.5%, respectively. Nonetheless, even with a free of charge PHC service enacted in 2006 the survey revealed that 26% of respondents did not visit a PHC facility [21].

Implementation of the household health expenditure survey by the USAID Primary Health Care Reform (PHCR) in March 2009 provides an opportunity to assess the progress made by Armenia in addressing the equity of access and affordability of healthcare since the previous such survey in 2006. The survey revealed that health expenditures were not evenly distributed. Over 30% of the richest quintile reported receiving outpatient services in 2008, but this fell to 20% - 25% of households in the next two income quintiles and only to 17% for the poorest households.

The main reason for not using health care was the lack of financial means - 78% in 2009 with comparison to 47% in 2007, self-treatment 43% (11% in 2007) and other (lack of trust in PHC providers’ qualifications and lack of time, etc.) 10% (11% in 2007) [22]. The household survey implemented by the USAID PHCR project revealed that in Yerevan, 44.5% of residents needing medical care went directly to pharmacies, instead of visiting a doctor and getting a proper prescription. Partially, this is to avoid making out-of-pocket payments to doctors still requiring fees for a consultation [23]. Out of pocket payments still accounted for more than half (51%) of total health expenditure in Armenia, whereas the public expenditure as a proportion of total health expenditure rose to 38.7% in 2008.

According to another, 2007 Integral Living Conditions Survey (ILCS), only about one third of respondents who reported being sick consulted a doctor. Among people consulting a doctor the share of those living in Yerevan was higher (38.9%) than those living in rural areas (28.9%) [24].

The 2009 ILCS data revealed, that the share of people who consulted a doctor varied also by poverty status. While 35.3 percent of the non-poor consulted a doctor for advice or treatment when sick, only 21.4 percent of the poor and 1.2 percent of the extremely poor did so. The majority of population (47.1%) consulted public medical facilities when sick and only insignificant part (5.4%) go to private clinics. At the same time, a significant share of the population (47.5%) consulted the drugstore staff.

Policlinics are among the most commonly visited public health care facilities (58%), since 2006 the entire population (without any social limitations) has been entitled to free ambulatory-polyclinic services, including physician fees, some of the most simple and rather not expensive primary lab and diagnostic tests, such as the cardiogram, X-ray, fluorography, the biochemical analysis and so on. Polyclinics were visited by 63% of Yerevan residents, compared to only 43% in the rural communities. From the view of the poverty status, policlinics are mainly visited by the very poor (75%) and poor (61%), while as hospitals and private facilities, including the expensive diagnostic centers are used mainly by the non-poor. The extremely poor never visited private medical facilities, even the healers. Diagnostic centers were visited by 97 percent of the non-poor and 3 percent of the poor.

In the bottom quintile of the population, only about one quarter of those reporting sickness received professional care, while over half of the sick in the wealthiest quintile received professional attention. The type and most probable quality of health care received also varied with income. The utilization of healthcare services in the poorest quintile was 25 times lower than the average utilization rate of these services (near $3.5 or AMD 1,283), whereas for the richest quintile this indicator was 4.2 times higher than the average [25].

Our study differs from the above-mentioned surveys by its aim, objectives and methods. The aim of our study was more specific and focused only on the health sector (health care services cost, utilization, satisfaction, etc.), while other surveys with more informative character were intended to inform the public about the living conditions and social situation in the country, described poverty trends nationally and by different socioeconomic, demographic and geographical classifications, or showed consumer expenditures on all types of services, including health. The purpose of this project was to explore the respondents’ awareness about free-of-charge medical services, the level of their satisfaction with provided medical care, the main reasons for the high level of unmet needs for health care, health inequalities and inequities. During our survey the information was collected using anonymous telephone interviews, while during other surveys face-to-face interviews with the household head or another knowledgeable adult member was used. We tried to contribute to the development of public health policy in Armenia by exploring the implications for delivering health services aimed at reducing health inequalities and inequities through action on the socio-economic determinants of health.

## Methods

We reviewed and analyzed the results of several household and patient surveys, specifically as they related to the utilization and accessibility of healthcare services, the reasons for not visiting a doctor, patient satisfaction with supplied services, and payments for healthcare. We conducted a literature review and analyzed national health data to understand if there was a correlation between the inequality in healthcare utilization/accessibility and health status in Armenia, and to identify the factors of their impact on the health of the population.

The survey of population health was conducted within the framework of the Armenian National Science & Education Fund (ANSEF) program in 2010 according to a specially developed questionnaire. The research was approved by the Institutional Review Board of Yerevan State Medical University (YSMU) after M. Heratsi (Armenian Unit of International Network of UNESCO Chair in Bioethics - Executive Committee; under protocol # 7 – 07.02.2010). Particular attention was given to the assessment of medical care satisfaction and to the assessment of the level of household expenditure on medical care. Stratified random sampling was used for the selection of respondents (1000 residents), with which there was an equal chance that each female or male respondent could be selected for inclusion in each stratum of our sample. The preliminary random selection was made among administrative divisions of Armenia on the basis of proportionate-to-population-size approach. We divided the respondents into groups or strata by urban/rural areas and the data was weighted by age and gender to bring the realized sample in line with target population parameters. Then, within each stratum, we randomly selected survey respondents.

A random sample of local residents was surveyed regarding the current usage of medical services, assessment of medical care satisfaction, household health expenditures and payments for healthcare, patient satisfaction with supplied services and perception of the healthcare system. Participants were also asked questions concerning:

demographic characteristics: sex, age (mainly 18-60+ years old), etc.;

education and occupation of respondents;

types of entities where the respondents work: public, private, NGO;

self-estimation of the basic aspects of health condition by the respondents;

estimation of local health care system activities by respondents;

risk factors and conditions influencing the health of population; social-economic, social-psychological, social-demographic, ecological, geographical, medical-organizational, etc.

Participation in the survey was voluntary. The data were collected using anonymous telephone interviews taking into account the fact that more than 90% of households have access to public telephone networks and 86% of Armenia's population uses mobile phones [26,27]. There were cases of refusal to participate in the interview and no answer from the respondent, however, explicit refusal by an eligible respondent was recorded only in 3.7% of the attempts. Therefore, overall, 1217 calls were made to complete 1000 questionnaires.

Calls were made to various regions based on the population size. Individual participants’ identifiers were not recorded. Quality control was managed through monitoring the interview process. Investigators were not purview to individual interviewee’s responses.

The ratio of respondents by gender was the following: female – 53,2%, male – 46,8%. The percentage ratio of age groups was as follows: 28% were between 18–25 years old; 32% - 26–45 years old; 24% - 46–60 years old; and 16% were over 60 years old. The main portion of respondents (79%) had higher education. According to their social - professional status respondents were - service employees −24%, specialists - 22%, students - 19%, retired - 10%, managers - 8%, entrepreneurs - 5%, housewives −9%, and the rest were unemployed −3%. The majority of respondents worked in the state structures (40%): budgetary or public organizations, 29% - in the private sector and rest of them in NGOs and other entities.

## Results

The analysis of the survey revealed that among the risk factors affecting the health of the population were noted: social-economic - 37.3%, social - psychological - 21.3%; social-demographic - 5.3%; ecological - 16%; geographical - 9.4% medical-organizational - 10.7%. According to the respondents the utilization of health care services was low, particularly in rural settlements. Low utilization of health care services in rural areas and to some extent also in small and medium sized cities is explained due to the lack of physical access to health care, remoteness from the nearest health facilities, dilapidated unheated buildings during extremely cold winters, often due to complete absence of pharmacies or their poor drug assortment: only few drugstores are enlisted for provision of free of charge drugs for vulnerable groups, which are either physically inaccessible, or many do not know their exact list and addresses. Some of the reasons also include lack of most basic supplies of equipment, a substantial cutback in hospital beds through mergers and closures due to the government optimization plan (whereas cities still have excess capacity). Besides, many respondents from these settlements reported that they prefer self - treatment or a visit to the healer.

According to our survey, 60% of respondents participating in the survey were sick, out of these 24% frequently were sick, 20% patients had chronic pathology and 16% were disabled. Affordability was the main concern for not using medical services, especially inpatient. 44% of the respondents could not afford and consequently did not have access to services; on a paid basis it was accessible to only 52% and only for 4% from all of the respondents eligible for BBP, it was accessible free of charge.

Results indicate utilization of health care is low even for Armenians who qualify for the government health care program BBP. This is in part because the government sets payment at rates that may not cover providers’ expenditures. As a result, BBP participants are pressured to give additional informal payments in exchange for health services as it is not based on real costs of services and thus contributes to unofficial or illicit payments. Moreover, all health services and drugs that are not included in BBP must be paid directly by the patient. For instance, in 2009 some 18 percent of population was entitled to the basic benefit package However, only 49% of the eligible population used health services under BBP, including 77% of the extremely poor, 51% of the poor, and 6% of the non-poor. Recipients of family benefit, who have used BBP in average spent monthly $31.4 (11401 AMD) per person, including $0.6 (214 AMD) under the BBP, informal payments amounted to $1.6 (587 AMD), $15.5 (5649 AMD) on medicaments, and the remaining amount channeled to official payments both at public and private medical facilities. As for the total number of population having used healthcare services (both paid and free of charge), the actual monthly amount per one patient totaled $56.8 (20625 AMD), including $2.5 (904 AMD) as informal payments, and $22.3 (8104 AMD) on medicaments, with the remaining amount spent as official payments for treatment [8].

The low affordability for using medical services can be also explained by the simple fact that only 8% of respondents received an income over $260 (over 100 000 AMD) per capita per month, while the overwhelming amount of respondents lived on an income of $80 (30 000 AMD) or less per capita per month, which is less than the minimum wage.

**Figure 1:**
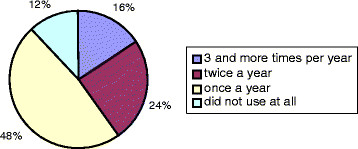
Health Services Usage by Survey Respondents in 2010.

Population awareness about free-of-charge medical services reached only 40%. The frequency that people utilized medical aid is shown in Figure 1. This figure shows that 12% of respondents did not use medical services at all, even when there was a need.

**Figure 2:**
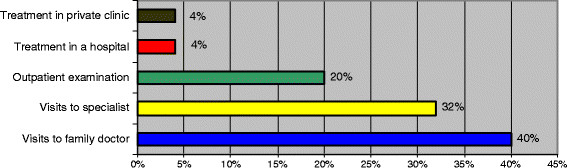
Frequency of visits by type of medical facility or specialist.

Many of them prefer the ostrich method: Better not know about our disease, than know about it and not be able to treat it because of lack of access to paid or more expensive services and drugs. The majority of the population visited public medical institutions when being sick and only 4% went to private clinics and hospitals (the low percentage of the last two kinds of medical services is conditioned by expensive treatments). Family practitioners and specialists of polyclinics were the major healthcare providers, as since 2006 the entire population (without any social limitations) has been entitled to free ambulatory-polyclinic services, but these are only some of the most simple and inexpensive services, the rest of health services that are not included in the list must be paid directly by the patient (Figure 2).

**Figure 3:**
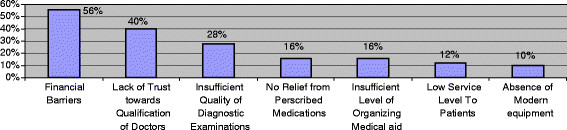
Distribution of population by main reasons for not seeking medical care.

More than 56% of respondents were not satisfied with provided medical care, and 36% were only partly satisfied. At the same time, the survey revealed that financial barriers and lack of trust towards doctors’ skill remain the main reasons for not seeking medical care and for high level of unmet needs for health care (Figure 3).

The financial barriers remain the main reason for high level of unmet needs for health care (56%). Results are not too surprising, considering the amount of the average monthly per capita monetary income. At the same time, the World Bank (WB) shows that the monthly size of “consumer's basket” in Armenia has made $112.8 (42158.7 AMD) and a “consumer's food basket” - $63.7 (23818.5 AMD) in the fourth quarter of 2010 [28]. It turns out, that the average monthly per capita monetary income was $84.4 (31553 AMD) i.e. 18% lower than the living wage - $103 (38487 AMD) [29,30]. Consumer expenditures in average monthly per capita monetary income were $ 76.7 (28646 AMD), including $21.6 (8082 AMD) or about 28.2% on all services, including health. At the same time, families with average monthly per capita income of $211 can be called “rich” [31]. Considering, that the index of income concentration (Gini index) in 2010 in Armenia was 0.36 [32], the picture will look even worse. Level of state funding for the core of the social sphere of Armenia is several times behind the world level. It is clear that from the insignificant amount for all services in household expenditures, it is impossible to allocate funds to meet even the minimum requirements for health services.

About 40% of respondents mentioned lack of trust in PHC providers and their qualifications. Trust has a strong emotional role in the success of the treatment efficiency. Treatment begins with the establishment of close personal contact between patient and doctor. In many cases, patients refuse to receive treatment due to mistrust in doctors. The main causes of distrust in the health care providers were because of technical incompetence and impersonal care, difficulty communicating with physicians, absence of incentive to treat patients with respect, provider’s focus on profit. These reasons were similar for men and women.

Technical incompetence was also indicated as a key aspect of distrust. It was explained by distrust of the respondents towards the doctors, who in their opinion, made a wrong diagnosis, gave the inappropriate treatment and provided the improper follow-up care.

Impersonal care was explained by many respondents as absence of care, empathy, patience and honesty. They shared about their experiences where the doctors barely talked to them and did not examine them, instead they immediately wrote a prescription, not even looking at them, if they didn't feel that they could get paid. Respondents also talked about how experimentation and beliefs about experimentation in the health care setting affected their views of the trustworthiness of doctors.

Some respondents expressed the view that doctors are there to make money, and many of them charge for various forms of unnecessary laboratory tests, procedures, medicines, services, etc. Payments are mostly out-of-pocket, contributing to the prosperity of the informal sector and undermining the principle of equity with respect to both financing and access. As to out-of-pocket costs for healthcare increase, requiring higher spending by households, the poor become much less likely to seek out professional care. Due to this cycle, the respondents group with the lowest level of income typically has the lowest health potential. This group also tends to have the most problems obtaining qualified medical assistance and medical goods, as use mostly the free of charge outpatient health services.

Only 57% of the sick (including many disabled and elderly with chronic diseases) in the poorest quintile refer to a doctor. This percentage could be much less if the ambulatory-polyclinic services since 2006 for most simple and inexpensive services have not been free. In contrast, 96% in the richest quintile used private clinics, inpatient and preventive services, which are not free. Moreover, their per capita expenses at diagnostic centers exceed total expenses made at other health institution.

The share of healthcare expenditures in total consumption for the richest quintile group was much higher compared to the poorest quintile group (9.1%and 0.1% respectively). The utilization of healthcare services in the poorest quintile is 17.1 times lower than the average utilization rate of these services (in AMD), whereas for the richest quintile this indicator is 5.8 times higher than the average. Hence, healthcare is increasingly considered a privilege for the elite, less and less available for the poorer part of the population.

## Conclusion

Thus, during the 1990s in Armenia both socio-economic situation and the policy responses led to an exacerbation of the crisis in the health care system with severe negative consequences for the population, especially in terms of equity and financial risk protection, unlike some other countries, for example, Kyrgyzstan and the Republic of Moldova, that had a clear set of policy objectives and a coherent approach to selecting policy instruments, and where the economic crisis was turned into a health reform opportunity [33].

A very important methodological conclusion can be derived from the trends for several national health indicators observed from 1990 to 2011. Utilization/accessibility to health care and the role of the state as a healthcare provider are open questions in Armenia.

The analysis of the surveys revealed that adverse outcomes of healthcare were observed in Armenia. On the one hand, the expenditures on medical services are high and continue to grow quickly, and on the other hand, access to the treatment is limited, and preservation of health becomes too expensive for a significant part of the population. The demand of health services and the level of utilization remain dramatically low, particularly in rural areas and among the poor. The way healthcare services supply is organized in Armenia shows social inequality in the access and utilization to such care. The qualitative and quantitative usage of medical services by the population is highly polarized which in its turn aggravates sharp social stratification and has an increasingly destabilizing social effect. As a result, the high level of poverty, and inequality of income distribution have had a negative impact on the health of the population and has also led to an increasing gap in the quality of medical care between the poor and the wealthy. The reliance on direct out-of pocket and informal payments, development of a grey market of paid medical services, are serious problems which have accumulated in the healthcare, pharmaceutical and medical technology sector, undermining the principle of equity with respect to both financing and access. In its turn, informal payments tend to penalize poor households and can have a significant impact on further access to health care services [34,35]. The financial barriers remain the main reason for not seeking medical care and for high level of unmet needs for health care. The expected number of medical visits is responsive to income. The greater the income, the greater the number of doctor visits.

The survey findings reiterate that lack of trust towards doctors’ skills will also remain as another major challenge for policy makers when reforming PHC provision. We believe that with the development of the general practice system, the patients’ choice of physicians will become increasingly meaningful. The right to choose a doctor should also enhance patients’ trust and quality of care, as physicians’ pay now depends on the number of patients, therefore, we can expect that they will invest more effort in ensuring patient satisfaction. In our view, the reform of primary health care, aimed at strengthening the link between patient numbers and physician pay, may encourage the movement of resources in this sector, improve the poor physical conditions in health posts and polyclinics, create more incentives for the staff to treat patients with respect and also promote the general practice model, all of which will enhance the standards of the sector and lead to greater patient satisfaction. An adequate strategy for training and re-training human resources for primary care needs to be developed in order to improve the trust of the general public in the quality of primary care providers. It is necessary to regain people's trust in doctors, to bring people back to the policlinics. Regaining trust is important for the sake of improving the results of patient satisfaction.

Despite the fact that practically almost all doctors and health institutions are de facto heavily involved in commercial delivery of health services, the private health sector in Armenia is still in need of further development. The establishment of private hospitals and clinics are still limited, because the private sector does not see for-profit health institutions as economically viable and the decline in demand for health services, make this market unattractive for private investment [36].

Given the radical decline in the availability of public revenues that had occurred by the mid-1990s in Armenia the initial focus of reforms was on trying to specify the BBP more precisely. This seemed rational; however the ability to enforce a package of entitlements depends critically on the system's ability to purchase them. This means that there must be a purchaser in place, supported by information systems that enable a link to be made between provider payment and clinical/patient data from providers. The existence of such systems makes it possible not only to declare the entitlements of the population, but actually to purchase them. Without them, the evidence suggests that the effort at formal rationing will be undermined by informal methods (such as informal payments in Armenia).

The reform experience also suggests some lessons with regard to what not to do, either in terms of policy or implementation. Many health systems face pressures to calculate the “true” or “real” cost of their benefits packages. This has been the case in Armenia (as stated above) and in several other countries (such as Ukraine and Georgia) [33]. Worse, fixing unit costs and contracting on that basis may actually inhibit restructuring reforms as the contractual price is likely to be also overstated and this will reduce the incentives for further downsizing. Another problem associated with the approach to benefits package design. Basically, a quite complex and a highly detailed package creates favorable conditions for provider manipulation, and in so doing can contribute to informal payments.

Starting the financing reform process with major modifications of the BBP is also politically dangerous, as the specification of entitlements and obligations is perhaps the most visible part of health financing policy to the population. If government is unable to deliver this (Armenia's experience is instructive here), the credibility of the entire reform process will be threatened. Social insurance may not be a panacea, particularly in a low-income setting. Armenia’s experience indicates the difficulty of reaching high levels of coverage using social insurance alone. There is a large level of informality and, in order to cover vulnerable groups, the reforms had to be adjusted to incorporate special targeting programs to reach those not covered by social insurance. The programs should be introduced at all points and levels for all strata and groups of population. In which it should be based on the principle of universal and equal access to health care. Otherwise, these healthcare programs will face a serious threat of scattering out and not serving their objective. Only strategically designed out-of-pocket payments - in conjunction with social insurance - can play an important role in ensuring sustainability of health care financing. They help moderate demand and ensure that poor and other vulnerable groups receive coverage for essential health care and protection from catastrophic expenditures.

We should strive for a system that covers everyone, to make the health system more responsive to the needs of the poor. Considering the issues of accessibility and utilization of health care services, as well as high prevalence of unofficial out-of-pocket payments, the growth of public spending in the medium to long term shall be among the top priorities of the national policy in the sector. However, apart from increasing public funds for health care, mainly hospital care and chronic disease medications, Armenia should explore alternative delivery methods for reaching out to the poor. In our view, in particular the support of private funding, which comes mainly from two sources - copayments, that vary with the services consumed and monthly contributions are also essential for a sustainable health system. The introduction of a co-payment mechanism for Armenia's predominantly public healthcare system in 2011 will increase the transparency of patient expenditure and as a consequence, to some extend, will reduce corruption and the widespread use of unofficial payments to medical employees, as well as successfully expand health care coverage. In its turn, the combination of a transparent payment system and additional funds generated by the co-payment mechanism will benefit healthcare finances and support growth for healthcare service providers, including drug makers.

Armenian government should make more deliberate efforts to improve the domestic pharmaceutical industry, reduce the nation's reliance on imported drugs and shift spending away from expensive imported medicines towards generic drugs, as the substitution of imported pharmaceuticals with domestically-produced medicines is an effective and reliable way for healthcare services cost reduction.

In conditions of widespread absence of pharmacies, especially in rural areas, we are of the opinion that, from the viewpoint of both awareness building and ensuring full access to drugs, it would be appropriate to delegate the responsibility for provisioning the mentioned drugs only to drugstores located in polyclinics and/or family doctors with the corresponding formalized agreement with polyclinics. They should be put in charge of distributing not only drugs provided free of charge and with privileged conditions, but also a certain quantity of drugs needed for emergency medical care. This measure can improve the state control over these resources and provide more efficient use of public funds, promoting access to drugs, particularly for vulnerable. This could be accomplished through public payments (monetary vehicles) that increase the amount of subsidies per individual to poor and lower income groups by implementing a compulsory health insurance system. In addition, the MoF to independent auditors should protect the budgetary allocations for basic health services by emphasizing the positive relationship between investments in health and poverty reduction.

It is essential in order to stop the downward spiral of healthcare utilization by reducing cost of services, eliminate informal payments to shadow operations in health care services and medical care sectors. In addition, keep financially strapped hospitals in low-income areas functioning with the help of special state funds and expand opportunities for the poor to obtain improved access and treatment.

Each country used a different set of policy instruments, but they exhibited important similarities in the process. However, for the effective reform process all of the key elements (problem definition, establishing policy objectives, choosing policy instruments, ensuring prioritization and sequencing, coordinating across functions, and so on) need to be defined, integrated, coordinated and managed in an objective way, incorporating evidence-based policy approaches.

There is no endpoint to the reform process, and good stewardship is essential in order to identify when changes are needed and to develop the appropriate responses. Regulatory and administrative capacity is critical to the success of reform, including successful expansion of health care coverage and must be backed up by a commitment to provide access to quality health care services. For instance, the development of public hospitals’ management capacity and the promotion of participatory management will lead to improvements in hospital performance.

International experience also has shown that а critical reform implementation step is the establishment of a new agency responsible for pooling funds and purchasing services, which typically goes hand-in-hand with the introduction of a new dedicated tax (Armenia is a notable exception, as the SHA here was established without the introduction of a new dedicated tax). Experience shows that many successful reformers began with the simultaneous introduction of a new dedicated (usually payroll) tax and a new agency responsible for pooling and purchasing. Simply creating the new agency, as it was in Armenia, is not enough to make it an effective agent of change. A new agency needs to be accompanied by measures to create or strengthen the purchasing function. In most countries, it was necessary even to start with a new institution outside of the MoH, with an off-budget status to overcome the rigidities of the inherited system.

Most transition of the countries introduced payroll or otherwise dedicated tax-funded compulsory health insurance funds in an effort to reverse the revenue decline experienced in the early transition period. The impact of this change on both the level of public revenues for health – and on wider policy objectives such as promoting universal financial protection and access to care – depended critically on the extent to which this reform was coordinated with corresponding changes in the level and flow of general budget funding, and on the coordination of these different sources of public funds via changes in pooling arrangements. However, the impact of introducing a dedicated tax for health on the level of funds is hard to discern due to concomitant underlying changes in fiscal context in these countries. For the CE countries that had a less severe economic transition, changes in the level of public revenues raised via dedicated taxes grew in line with changes in the overall economy, similar to overall public revenues. For the more severely affected countries that also introduced payroll tax, the level of funds raised was not great. In each case, however, corresponding reforms with regard to the allocation of general budget revenues to health were of critical importance.

The examples are the positive experiences of the Czech Republic and the Republic of Moldova, which introduced defined central budget transfers to their insurance funds on behalf of specific non-contributing groups of the population. The evidence from the region suggests that it is essential that clear commitments for budgetary funds are designed as an integral part of the compulsory health insurance introduction, in order to avoid offsetting revenue declines (and also to promote universality), and further suggests that such commitments are more likely to be implemented when the source of budget funds is the central rather than decentralized levels of government.

An efficient tax collection mechanism and a low rate of tax evasion have also helped ensure that the necessary resources will be available to finance public spending. The current tax laws in Armenia should be improved, to give more incentives to the employer to provide health insurance to its employees. Besides, the tax should not be high, especially in low- income countries (for example, in Kyrgyzstan it was - and remains - only 2% of payroll), but it seems to be necessary as a means to create new institutional arrangements that generate opportunities to drive broader health financing reforms.

Many of the countries that introduced compulsory health insurance also changed the nature of entitlement from citizenship to contribution. In doing so, they faced the problem of creating explicitly uncovered population groups for the first time (taking into consideration that prior to 1990 they had universal coverage). Related to this was the possibility of introducing a new form of fragmentation: different systems for the insured and uninsured parts of the population. Creating such parallel systems could have contributed to overall efficiency and equity problems, as has frequently been the case in many low and middle-income countries that introduced compulsory health insurance in contexts in which a large share of the population is not employed in the formal sector [37-40]. From the start, however, most Central Europe, Eastern Europe, the Caucasus and central Asian (CE/EECCA) countries that introduced compulsory health insurance simultaneously introduced measures to fund the coverage of non-contributing population groups.

There is a need to create a strategy to reduce fragmentation and align incentives. Critical to this is the strengthening of purchasing mechanisms in the system and altering the flow of general budget funds from subsidizing supply to subsidizing the purchase of services on behalf of the population. Related to this, in their turn, are reforms in pooling to enable reduced fragmentation or at least explicit coordination of the use of funds from different public sources.

The development of information systems is essential, as investments in computer systems have allowed financial managers to monitor flow of revenues daily, have facilitated the implementation of new payment mechanisms to enhance provider efficiency and performance. Despite some investments in information technology, Armenia currently faces extensive demands in this area. For Armenia, it would be wise to learn from the reform experience of other countries, and to accelerate its reform since experience in health care reform in some of them, especially in countries with transition economy with excess capacity and reduced fiscal space could be a good lesson for the Armenian government. For example, the Kyrgyz Republic for a country of its income level has a well-developed health information system that facilitated policy development, especially prospective provider reimbursement, based on enrollment at primary care facilities, hospital admissions, and outpatient utilization [41].

A further important factor in the successful implementation of the reform process could be а high level of co-ordination and co-operation between the government and all the key international and bilateral donors working in the health sector in Armenia. This joined-up thinking will help ensure that improving access to health services remains at the centre of policy development.

## Endnotes

The article is written within the framework of the Armenian National Science & Education Fund (ANSEF) program.

## Abbreviations

AMD, Armenian dram ($1=373, 66 drams); ANSEF, Armenian National Science & Education Fund; BBP, Basic Benefit Package; CIS, Commonwealth of Independent States; CE, Central Europe; CE/EECCA, Central Europe, Eastern Europe, the Caucasus and Central Asia; ECA, European and Central Asia; GDP, Gross Domestic Product; ILCS, Integrated Living Conditions Survey; MoF, Ministry of Finance; MoH, Ministry of Health; NSS RA, National Statistical Service of the Republic of Armenia; PHC, Primary Health Care; PHCR, Primary Health Care Reform; SHA, State Health Agency; SU, Soviet Union; UNESCO, United Nations Educational, Scientific and Cultural; UNICEF, United Nations International Children's Emergency Fund; USAID, United States Agency for International Development; WHO, World Health Organization; WB, World Bank; YSMU, Yerevan State Medical University.

## Competing interests

The authors declare that they have no competing interests.

## Authors’ contributions

TT conceived the idea, collated, analyzed and summarized the data and wrote the paper. She was principal investigator of the study and drafts the manuscript. LM helped in collecting the data. Co-author read and approved the final manuscript.

## Authors’ information

Tamara Tonoyan is a Ph.D. of Economics, Associate Professor of Health Economics at the Yerevan State Medical University (YSMU) after Mkhitar Heratsi and Head of the Department of Health Policy, Economics and International Relations at the National Institute of Health, Ministry of Health, Republic of Armenia (NIH MH RA).

Lusine Muradyan is a Doctor of Medical Science, Associate Professor at the Yerevan State Medical University (YSMU) after Mkhitar Heratsi, Republic of Armenia.
